# Perceived Impact of COVID-19 on Older Adults’ Mental Health and Barriers to Mental Health Care

**DOI:** 10.1093/geroni/igab046.2690

**Published:** 2021-12-17

**Authors:** Grace Caskie, Madison Tschauner, Eve Root

**Affiliations:** 1 Lehigh University, Bethlehem, Pennsylvania, United States; 2 Lehigh University, Philadelphia, Pennsylvania, United States

## Abstract

COVID-19 disproportionately impacted older adults in terms of fatalities, but also increased stress, isolation, and loneliness (Chen, 2020). We examined older adults’ anxiety, depression, and barriers to mental healthcare during the COVID-19 pandemic and their perceptions of these variables prior to the pandemic. Further, we explored whether any perceived changes differed based on geographical location (rural, suburban, urban). Data were collected online between mid-June and mid-July of 2020 from 244 individuals aged 65-82 years (M=68.3, SD=3.5). The sample was primarily White (91%) and female (60%); most (n=119) lived in suburban settings, with 63 in urban and 60 in rural settings. Repeated-measures ANOVAs at alpha=.01 showed that depressive symptoms, measured by the CESD-10 (p<.001), and anxiety symptoms, measured by the GAD-7 (p<.001), increased during the pandemic as did mental healthcare barriers related to transportation (p=.004) and beliefs that depression is a normal part of aging (p<.001). Only transportation concerns differed based on where older adults lived; those in rural (p<.001) and urban (p=.004) settings reported greater transportation barriers than those in suburban settings. No differences over time were found for barriers related to help-seeking (p=.403), stigma (p=.156), knowledge/fear (p=.180), finding a therapist (p=.030), ageism (p=.302), psychotherapist qualifications (p=.265), physician referrals (p=.207), or finances (p=.818). These findings highlight the impact of COVID-19 on older adults’ perceptions of changes in their psychological well-being as well as their experience navigating mental health services.

